# A review of urinary angiotensin converting enzyme 2 in diabetes and diabetic nephropathy

**DOI:** 10.11613/BM.2019.010501

**Published:** 2018-12-15

**Authors:** Akankwasa Gilbert, Jianhua Liu, Guixue Cheng, Changjuan An, Kabuye Deo, Abalinda Mary Gorret, Xiaosong Qin

**Affiliations:** 1 Department of laboratory medicine, Shengjing Hospital of China Medical University, Shenyang, People’s Republic of China; 2 Department of laboratory medicine, First Teaching Hospital of China Medical University, Shenyang, People’s Republic of China; 3 Department of medical laboratory science, Mbarara University of Science and Technology, Mbarara, Uganda

**Keywords:** angiotensin converting enzyme, diabetic nephropathy, mRNA

## Abstract

Urinary angiotensin converting enzyme 2 (ACE2) is significantly increased in diabetes and diabetic nephropathy. While studies on its clinical significance are still underway, its urinary expression, association with metabolic and renal parameters has been in the recent past considerably studied. The recent studies have demystified urine ACE2 in many ways and suggested the roles it could play in the management of diabetic nephropathy. In all studies the expression of urinary ACE2 was determined by enzyme activity assay and/with the quantification of ACE2 protein and mRNA by methods whose reliability are yet to be evaluated. This review summarizes recent findings on expression of urinary ACE2, examines its relationship with clinical parameters and highlights possible applications in management of diabetic nephropathy.

## Introduction

**Figure 1:**
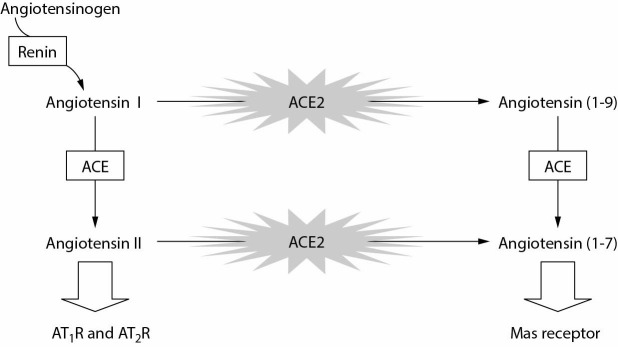
Schematic representation of the nephroprotective mechanism of angiotensin converting enzyme 2 and angiotensin (*1*-*7*). ACE2 - angiotensin converting enzyme 2. ACE - angiotensin converting enzyme. AT_1_R - angiotensin type (*1*) receptor. AT_2_R - angiotensin type (*2*) receptor.

Angiotensin converting enzyme 2 (ACE2) is an intra-renal component of the renin-angiotensin system (RAS) that significantly influences the pathogenesis of diabetic nephropathy (*1*-*3*). It alleviates vasoconstriction, fibrosis, oxidative stress, water and salt retention promoted by angiotensin II. Angiotensin converting enzyme 2 hydrolyses angiotensin II and angiotensin I; generating angiotensin (*1*-*7*) and angiotensin (*1*-*9*) respectively (Figure 1). Acting through the ACE2 - angiotensin (*1*-*7*) – Mas axis, angiotensin (*1*-*7*) endogenously counteracts the deleterious effects of angiotensin II and affords protection to kidneys.

Diabetic nephropathy is a kidney disease that arises as a complication of diabetes type 1 or type 2. It is a cause of end stage renal disease (ESRD) in developed and developing countries and is associated with a risk of cardiovascular diseases. The prevalence of diabetes and diabetic nephropathy is on an increasing trend especially in the population below 18 years (*4*). The incident rate of kidney failure attributed to diabetes has remained high in spite of the advances in diagnosis and treatment of diabetic nephropathy; in the United States, 120,688 new cases of ESRD were reported in 2014 representing a 1.1% increase from 2013 (*5*). Also, diabetes accounted for 44% of patients treated for ESRD (*6*). It takes 10-20 years for a patient with diabetic nephropathy progress to ESRD.

The first clinical sign of diabetic nephropathy is albuminuria in the range of 30 - 300 mg/day, which gradually increases as the renal function declines. Since 1980’s, albuminuria has been a diagnostic standard for diabetic nephropathy and is still being considered a risk factor for renal complications among the diabetics (*7*-*9*). Lately, however, its suitability for diagnosis and prognosis has been questioned as it emerged that about 50% of diabetic patients with renal impairment remain normoalbuminuric (*10*). Albuminuria is preceded by biomarkers of both glomerular and tubular dysfunction, suggesting albumin as a late indicator of diabetic kidney disease. For instance urinary cystatin C, kidney injury molecule-1, neutrophil gelatinase associated lipocalin, podocin and nephrin were reported to precede the onset pathological albuminuria (*11*-*14*). These observations have warranted a search for new diagnostic and prognostic biomarkers. A number of biomarkers including urine transferrin, urine retinol binding protein (RBP), serum osteopontin and urine ACE2 have already been proposed, their diagnostic and prognostic potentials await evaluation (*15*, *16*).

Being significantly increased among the normoalbuminuric diabetics, urinary ACE2 holds both diagnostic and prognostic potentials worth investigating (*16*). Moreover intra-renal ACE2 has profound effect on development of diabetic kidney disease (*1*, *2*, *17*). It is abundantly expressed on the brush border of proximal renal tubular epithelium from where it is proteolytically cleaved within its ectodomain and released into urine.

Since its identification in early 2000’s, the value of urinary ACE2 in the management of diabetic nephropathy has never been appreciated. We explored recent studies and examined relationships between urinary ACE2 and clinical parameters to highlight possible applications in management of diabetic nephropathy. This review summarizes what has already achieved and sets a stage for new studies.

## The shedding mechanism and detection techniques for urinary angiotensin converting enzyme 2

Urinary ACE2 originates mainly from the proximal renal tubular epithelium where is abundantly expressed. The urinary shedding of kidney-expressed ACE2 is a phenomenon that occurs in both health and disease. Although the shedding occurs in health and various kidney diseases, its occurrence is greatly enhanced in diabetes and is mediated by a disintegrin and metalloproteinase 17 (ADAM 17) whose renal expression is up regulated by high glucose concentration (*16*, *18*-*28*). The glucose-augmented shedding of ACE2 was found to be attenuated by administration of insulin and rosiglitazone (*23*, *24*).

The presence of ACE2 in urine is determined by the urine assays for three expression forms namely; ACE2 protein, ACE2 mRNA and the enzymatic activity. Angiotensin converting enzyme 2 protein is quantitatively determined by enzyme linked immunosorbent assay (ELISA) and immunoblotting while the enzyme activity and mRNA are quantified by fluorometry and real time polymerize chain reaction (RT-PCR), respectively (*16*, *18*-*26*, *29*-*32*).

The quantification of urinary ACE2 requires freshly collected or preserved urine samples. Pending analysis, collected urine samples can be kept at - 80 ^o^C for a period less than 2 months to avoid deterioration. The measurement of ACE2 by ELISA, immunoblotting and fluorometry requires urine supernatant while RT-PCR requires urine pellets. ELISA kits, polyacrylamide gel and ACE2-specific substrates required for ACE2 ELISA, immunoblotting and fluorometry are all commercially available. The commonly used ACE2 – specific synthetic substrates are *o*-amino-benzoic acid-Ser-Pro-Tyr(NO2)-OH and Mca-APK(Dnp) (*16*, *18*, *21*, *23*-*26*, *31*). Laboratory procedures for ACE2 ELISA, immunoblotting, fluorometry and RT PCR were detailed by Xiao *et al.* (*21*).

Although ELISA, real time RT-PCR and fluorometry have for long been used to measure urinary ACE2 mainly for research purposes, their performances are not yet evaluated. A few inconsistencies regarding the use of these assay techniques have been encountered but not formally reported. Measured by ELISA, the level of urine ACE2 was found significantly decreased upon induction of hyperglycaemia among the previous euglycaemic patients (*31*). This observation suggested ELISA as insensitive to ACE2 in hyperglycaemia, it still waits to be resolved. Furthermore, the level of ACE2 protein was found increased in diabetic transplant recipients as the mRNA remained unchanged in the same subjects under similar conditions (*21*). In spite of the difference between ACE2 protein and ACE2 mRNA expression forms, this observation suggested that the two methods are incomparable.

The urinary ACE2 assay could be affected by different factors some of which are still lying unidentified. Therefore, there is a need to establish the reliability of these methods and identify conditions that best suit their applications. At present, there is no published information regarding their comparability, advantages and limitations. Their performances ought to be evaluated not only for future adaption in the clinical practice but also research in which they are already being used.

## The expression of urinary angiotensin converting enzyme 2 in diabetic subjects with and without nephropathy

Diabetic nephropathy is defined as structural or functional abnormality of kidneys attributed to diabetes. The diagnostic criteria for diabetic nephropathy is an estimated glomerular filtration rate (eGFR) < 60 mL/min/1.73m^2^ and a urine albumin concentration > 30 mg/day according to the consensus conference on chronic kidney disease and diabetes by the American Diabetes Association and the Nephrology and the National Kidney Foundation (*33*). This is also in accordance with the 2012 Kidney Disease: Improving Global Outcomes (KIDGO) clinical guidelines for the management of chronic kidney disease (CKD) (*34*).

A survey of the recent studies indicated that urine ACE2 is significantly expressed in the diabetic subjects compared to the non-diabetic. The study findings regarding expression of ACE2 protein, enzyme activity and mRNA are as summarized in Table 1.

**Table 1:** Summary of expression patterns of urinary angiotensin converting enzyme 2 protein, enzyme activity and mRNA in diabetes and diabetic kidney disease

**Condition**	**Year** **(reference)**	**Study type** **(N)**	**Expressional forms of angiotensin converting enzyme 2**		
			**Protein** **(methodology)**	**Activity** **(methodology)**	**mRNA** **(methodology)**
Type 1 diabetes	2017 (18)	Human (194)	↑ (ELISA)	↑ (fluorometry)	ND
Diabetic nephropathy	2015 (19)	Human (72)	↑ (ELISA)	ND	ND
Type 2 diabetes	2013 (20)	Human (412)	↑ (ELISA)	ND	ND
Diabetic renal transplant recipients	2012 (21)	Human (38)	↑ (ELISA, immunoblotting)	↑ (fluorometry)	_ (RT-PCR)
Diabetic nephropathy	2011 (22)	Human (38)	↑ (immunoblotting)	ND	ND
Diabetic nephropathy	2008 (32)	Human (50)	ND	ND	NR (RT-PCR)
Type 2 diabetes	2016 (29)	Human (75)	NR (ELISA)	ND	ND
Type 2 diabetes	2015 (16)	Human (132)	↑ (ELISA, immunoblotting)	↑ (fluorometry)	ND
Diabetic nephropathy	2015 (30)	Human (31)	NR (ELISA)	ND	ND
Diabetic nephropathy	2013 (23)	Animal (NR)	↑ (immunoblotting)	↑ (fluorometry)	ND
Diabetic nephropathy	2014 (24)	Animal (NR)	↑ (immunoblotting)	↑ (fluorometry)	ND
Diabetic nephropathy	2014 (25)	Animal (NR)	ND	↑ (fluorometry)	ND
Type 2 diabetes	2013(26)	Animal (NR)	↑ (immunoblotting)	↑ (fluorometry)	ND
Type 1 diabetes (clamped euglycemia)	2014(31)	Human (58)	↑ (ELISA)	↑ (fluorometry)	ND
NR - not reported. ND - not determined. ↑ - significantly increased expression. _ - unchanged expression. ELISA - enzyme linked immunosorbent assay. RT-PCR - real time polymerize chain reaction.					

The protein expression and enzyme activity were significantly increased among diabetic patients (*18*-*20*, *22*). The diabetic renal transplant recipients also excreted higher amounts of urine ACE2 protein compared to their non-diabetic counterparts (*21*). In study form Xiao *et al.* all three expression forms were studied but unlike the mRNA which remained unchanged, the enzyme activity and protein expressions were significantly increased (*21*).

The assay of activity and protein were preferred methods for studying urinary expression of ACE2 in majority of the studies (*18*, *21*, *23*-*26*, *31*). The expression of ACE2 by mRNA was studied, but less frequently (*21*, *32*). Whenever concurrently determined, the enzyme activity and ACE2 protein were found similarly expressed as both increased significantly among the diabetic subjects (Table 1). In the study where ACE2 mRNA was reported, it remained unchanged in spite of the significant increase in other expression forms of urinary ACE2 (*21*). No discrepancies were observed between the expression of ACE2 protein and the enzyme activity except in clamped hyperglycaemia when the protein expression was suppressed (*31*). The observed discrepancy could have arisen from poor sensitivity due to glycosylation of ACE2. Glycosylation renders ACE2 insensitive to ELISA potentially causing a mismatch between enzyme activity and protein level.

Due to the consistence in expression of ACE2 protein and enzyme activity as observed in majority of the studies, it is tempting to conclude that the two are more reliable than the measurement of ACE2 mRNA. The reliability of these expressional forms of ACE2 need to be evaluated in well-designed studies with representative samples sizes as the reviewed studies on ACE2 mRNA were too few for such a conclusion.

It was evident however, on the basis of the reviewed studies, that the assay of protein and enzyme activity gives comparable results (Table 1). As such, both can be relied upon as indices of urinary ACE2 considering their concurrent increased expression among diabetic patients. It is thus irrational to measure both activity and protein *per* current practice. The assay of either protein or enzyme activity would suffice saving time and other resources. It should therefore be adopted.

It also became apparent in this review that the increase in urinary ACE2 is not specific to diabetes or diabetic nephropathy. Urinary ACE2 was also significantly increased in the non-diabetic kidney diseases although highly augmented in diabetes (*19*, *22*). The non-diabetic kidney diseases have of recent posed a challenge to the management of diabetic nephropathy as they have been reported to occur with diabetic nephropathy. The most important non diabetic kidney diseases known to complicate diabetic nephropathy include immunoglobulin A nephropathy and membranous nephropathy (*35*). The renal pattern of ACE2 in immunoglobulin A nephropathy was by coincidence found identical to that of diabetic nephropathy suggesting that ACE2 influences the progression of diabetic and non-diabetic kidney diseases in a similar manner (*36*). This notwithstanding, the expression of urine ACE2 in immunoglobulin A and membranous nephropathies has never been studied. Accordingly, future studies should also examine and elucidate the potential that urinary ACE2 may hold for the diabetic nephropathy-complicating non diabetic renal diseases.

## Associations of urinary angiotensin converting enzyme 2 with renal and metabolic parameters

Deranged metabolic pathways of glucose bring hyperglycaemia, the underlying cause of diabetic nephropathy. Activation of some glucose metabolic pathways causes oxidative stress and the accumulation of advanced glycated end products which are injurious to the kidneys.

Little is known how urinary ACE2 relates with the routinely used parameters to evaluate the metabolic status and renal function of patients with diabetic nephropathy. We reviewed recent studies and analysed the relationship between urinary ACE2 and the concentration of each of the following serum/urine metabolites/biomarkers; glucose, triglycerides, total cholesterol, albumin creatinine ratio (ACR), total proteinuria, serum creatinine, urinary nephrin, liver type fatty acid binding protein (L-FABP) and the eGFR. The findings are as summarized in Table 2.

**Table 2:** Associations of urinary angiotensin converting enzyme 2 with metabolic and renal parameters

**Condition**	**Year (reference)**	**Study type** **(N)**	**Findings**
Type 2 diabetes	2015 (16)	Human (132)	1. Urinary ACE2 was significantly increased in both albuminuric and non albuminuric diabetic patients. 2. Significant correlation was found between urinary ACE2 and each of the following parameters; fasting blood glucose, triglycerides, total cholesterol and glycated haemoglobin. 3. Urinary ACE2 was independently predicted by glycated hemoglobin.
Type 1 diabetes	2017 (18)	Human (194)	1. No significant correlation was found between urinary ACE2 and renal parameters (ACR and eGFR)
Diabetic nephropathy	2015 (19)	Human (72)	1. Significant association was found between urinary ACE2 and each of urinary ACR and L-FABP. 2. A significantly high level of urinary ACE2 was found at CKD stage 4.
Type 2 diabetes	2013 (20)	Human (412)	1. A positive association was found between urinary ACE2 and each of the following parameters; ACR, serum creatinine, triglycerides and blood glucose.
Diabetic renal transplant recipients	2012 (21)	Human (38)	1. A significant association was found between ACR and urinary ACE2 protein but not enzyme activity.
Diabetic nephropathy	2008 (32)	Human (50)	1. Significant correlation was found between urinary ACE2 mRNA and proteinuria.
Type 2 diabetes	2016 (29)	Human (75)	1. A positive association was found between urinary ACE2 and urine nephrin.
Diabetic nephropathy	2015 (30)	Human (31)	1. A positive association was found between urinary ACE2 and L-FABP but not ACR and eGFR among patients being treated with olmesartan.
Diabetic nephropathy	2013 (23)	Animal (NR)	1. Urinary ACE2 was attenuated by rosiglitazone. 2. Significant correlation was found between urinary ACE2 and each of the following parameters; plasma glucagon, plasma triglycerides, blood glucose and urine albumin.
Diabetic nephropathy	2014 (24)	Animal (NR)	1. Insulin treatment decreased urinary ACE2 together with urine albumin, serum creatinine, blood glucose, serum glucagon and triglycerides in diabetic mice. 2. Significant correlation was found between urinary ACE2 and each of the following parameters; urine albumin, serum creatinine, serum triglycerides, glucagon and blood glucose.
Diabetic nephropathy	2014 (25)	Animal (NR)	1. Urinary ACE2 was attenuated by daily exercise with and without metformin treatment. 2. A significant correlation was found between urine ACE2 and urine albumin in exercising mice with and without metformin treatment.
Type 2 diabetes	2013 (26)	Animal (NR)	1. Urinary ACE2 was decreased by insulin treatment.
Type 1 diabetes (clamped hyperglycaemia)	2014 (31)	Human (58)	1. Induction of hyperglycaemia suppressed the expression of urinary ACE2 protein levels but not the enzyme activity.
ACR - albumin creatinine ratio. NR - not reported. ACE2 - angiotensin converting enzyme 2. L-FABP - liver type fatty acid binding protein. CKD - chronic kidney disease. eGFR - estimated glomerular filtration rate.			

Presently, there are no reliable means of identifying diabetic patients at risk of developing diabetic nephropathy. Although albuminuria is currently being used for this purpose, it serves no value among the 50% diabetic patients who remain normalbuminuric but develop renal insufficiency (*10*). Accordingly, new biomarkers with better performance are needed. We reviewed and analysed the plausibility of having urinary ACE2 as a diagnostic and prognostic biomarker of diabetic nephropathy. We assessed its potential by how it relates with the established metabolic and renal parameters among the diabetic patients with and without nephropathy.

All studies reported a positive association between urinary ACE2 and all/ majority of the renal and metabolic parameters except one (*16*, *18*-*21*, *23*-*25*, *29*, *30*, *32*). The relationships ranged strong to weak perhaps depending on the severity diabetes and the degree of renal impairment.

Angiotensin converting enzyme 2 was found to be associated with urinary albumin and its concentration significantly increased in both albuminuric and non albuminuric patients (*16*, *19*-*21*). It was also positively associated with nephrin and L-FABP in diabetic patients (*19*, *29*).

Albumin, nephrin and L-FABP are renal parameters of whose presence in urine is indicative of renal dysfunction. Urine albumin is currently used in diagnosis and prognosis diabetic nephropathy while urinary nephrin and L-FABP are indicators of glomerular and tubulointerstial injury that occurs in the early stages of kidney disease. Like urinary ACE2, both nephrin and L-FABP were significantly increased among normoalbuminuric patients, positively correlated with proximal tubular dysfunction and negatively associated with eGFR (*14*, *37*, *38*). These observations suggest that urinary ACE2 rises in early stages of diabetes and could reflect the state of kidney function, an attribute of a good diagnostic and prognostic biomarker. The prognostic potential of urinary ACE2 was further underpinned by a recent study in which urinary ACE2 was listed among biomarkers that could predict the progression of diabetic renal disease in the early stages (*39*).

Urinary ACE2 was found associated with metabolites such as triglycerides, total cholesterol and glucose in diabetic patients (*16*, *20*). Metabolic abnormalities of these metabolites are associated with increased risk of CKD (*40*). Of all metabolites, glucose was the most studied in relation to urinary expression of ACE2. There is an increasing body of evidence implicating glucose in the urinary shedding of ACE2. Urinary ACE2 was significantly attenuated by insulin, rosiglitazone and metformin with/without exercise, the agents known to correct high blood glucose concentrations (*23*-*26*). Urine ACE2 was significantly correlated with glycated haemoglobin, a product of elevated blood glucose (*16*). The association of urine ACE2 with blood glucose was noteworthy as it is only glucose that has so far been shown to influence the renal expression of ADAM17, a protease responsible for the shedding of ACE2 (*24*, *27*). It is therefore evident that urinary ACE2 is dependent on blood glucose, a metabolite whose deranged metabolism underlies diabetic nephropathy.

We also reviewed recent studies for associations of urinary ACE2 with serum creatinine and eGFR, the parameters used in staging of CKD. Urinary ACE2 was associated with serum creatinine and advanced stages of diabetic nephropathy in spite of the insignificant correlation with eGFR (*18*, *20*, *24*, *30*). The concentration of ACE2 was found significantly higher in diabetic patients at CKD stage 4 (defined as eGFR = 30 mL/min/1.73m^2^), suggesting a possible role in the staging of chronic diabetic kidney disease (*19*). Currently, the staging of CKD is done in accordance with KDIGO 2012 clinical practice guideline for the evaluation and management of chronic kidney disease. It makes use of eGFR calculated with the Chronic Kidney Disease-Epidemiology Collaboration (CKD-EPI) equation whose performance was found better than other equations including the Modification of Diet in Renal Disease (MDRD) (*41*-*43*).

The estimation of eGFR, however, is still problematic because it relies on serum creatinine which is subject to vary according to age, sex and muscle mass among patients. A search a better method of staging CKD is therefore warranted.

Taken together, the findings of this review suggest urinary ACE2 as a potential diagnostic and prognostic biomarker of diabetic nephropathy.

While caution was taken to only review authentic studies, it should be noted that some of the studies included did not comply with KDIGO 2012 clinical practice guidelines for evaluation and management of CKD while reporting urine albumin and eGFR. Also, some of the reviewed studies were conducted with small sample sizes. ACE2 was found correlated with both renal and metabolic parameters, these shortcomings nonetheless. Well-designed clinical studies are needed to further elucidate the potential that urinary ACE2 holds for diabetic nephropathy.

## Effect of renal therapy on expression of urinary angiotensin converting enzyme 2

Renin-angiotensin system blockade is an approved therapy for the management of diabetic nephropathy. Renin-angiotensin system blockers used in the treatment diabetic nephropathy include angiotensin converting enzyme inhibitors (ACEIs) and angiotensin II receptor 1 blockers (ARBs) (*44*, *45*). Like ACE2, these agents retard the progression of diabetic nephropathy by suppressing deleterious effects of angiotensin II. However, the effects of RAS blockers on urinary expression of ACE2 is not well appreciated and it is not known whether changes in urine expression patterns ACE2 are reflective of their therapeutic effect. We reviewed recent studies and examined the effect of RAS blockade on urine expression of ACE2. The findings are as reported in Table 3.

**Table 3:** Effect of renal therapy on expression of urinary angiotensin converting enzyme 2

**Condition**	**Year (reference)**	**Study type (N)**	**Findings**
Diabetic nephropathy	2011 (22)	Human (38)	1. The level of urine ACE2 was not affected by the use of angiotensin-converting enzyme inhibitor or angiotensin receptor blockers.
Diabetic nephropathy	2008 (32)	Human (50)	1. Urine expression of ACE2 mRNA was not affected by treatment with ACEI and ARB.
Type 2 diabetes	2016 (29)	Human (75)	1. There was no significant difference in the level of urine ACE2 protein between the patients treated with RAS blockers and the untreated ones.
Type 2 diabetes	2015 (16)	Human (132)	1. RAS inhibitors attenuated the enhancement of urine ACE2 among hypertensive diabetics. 2. Urine ACE2 was independently predicted by treatment with RAS inhibitors.
Diabetic nephropathy	2015 (30)	Human (31)	1. Urinary ACE2 was significantly increased by Olmesartan, an angiotensin II type I receptor blocker.
Type 2 diabetes	2013 (26)	Animal (NR)	1. The activity level of urinary ACE2 was not altered by telmisartan and captoril.
NR - not reported. ACEI - angiotensin converting enzyme inhibitor. ARB - angiotensin receptor blocker. RAS - renin-angiotensin system. ACE2 - angiotensin converting enzyme 2.			

It was observed in most of the studies that ACEIs and ARBs did not alter the activity, protein and mRNA levels of urine ACE2. Although this was the case in almost all studies, a few reported otherwise (*22*, *26*, *29*, *32*). For instance the use of Olmesartan, an ARB was reported to increase the urine expression of ACE2 while the use of RAS inhibitors attenuated the augmented expression of ACE2 among the hypertensive diabetics (*16*, *30*). Although the decrease in expression of ACE2 caused by RAS inhibitors in the Liang *et al.* study was significant enough to serve a measure therapeutic effect, it only occurred among the hypertensive diabetics (*16*). This observation conflicted with the Abe *et al.* study in which Olmesartan increase the urine expression of ACE2 (*30*).

It is therefore plausible to speculate that RAS blockers possess functional differences which, perhaps, are responsible for the varied effects on urinary ACE2. Moreover some functional differences were reported with the use of ARBs (*46*).

Taken together, these findings suggest that the urine ACE2 as not an ideal parameter for assessing the therapeutic effect of RAS blockade therapy in diabetic nephropathy. The effects of RAS blockers on expression of urinary ACE2 need to be further investigated.

## Conclusions

Angiotensin converting enzyme 2 was expressed in urine of diabetic and non-diabetic subjects at the level of protein and mRNA. Being an enzyme, ACE2 exhibited a catalytic activity the assay of which was also used to determine its urinary expression. The quantification of ACE2 protein and the assay of enzyme activity were the preferred methods of studying the expression of urine ACE2. In most studies, ACE2 protein and enzyme activity were concurrently measured. These studies yielded comparable results for the enzyme activity and protein expression but not ACE2 mRNA whose expression conformed to none of the two. The enzyme activity and protein expression were significantly increased in diabetes, unlike mRNA whose expression remained unchanged. The utility of ACE2 protein and enzyme activity was evident and supported by many studies while that of ACE2 mRNA was less evident and supported by few studies. The urine expression ACE2 mRNA should therefore be interpreted separately and cautiously as it may not represent the enzyme activity and protein expression of ACE2. In studying urinary of ACE2, the assay of either ACE2 protein or enzyme activity but not both is recommended since the two expressional forms associate well in diabetics.

The urine expression of ACE2 was reasonably associated with renal parameters including ACR, eGFR, total proteinuria, serum creatinine, urinary nephrin and L-FABP. Urinary ACE2 was found correlated with ACR, a diagnostic and prognostic biomarker of diabetic nephropathy. The same was observed with eGFR, a parameter used for staging of CKD. This was observed in several studies including those in which ACR and eGFR were not reported according to the KDIGO 2012 clinical practice guideline for the evaluation and management of CKD. Taken together, the studies provide considerable evidence in support of urinary ACE2 as a surrogate biomarker of albuminuria. Accordingly, future studies should aim at elucidating the diagnostic and prognostic potentials that urinary ACE2 holds for diabetic nephropathy.

Urinary ACE2 was significantly associated with metabolites including blood glucose, cholesterol and triglycerides. The association with glucose was noteworthy as blood glucose alone influenced the renal expression of ADAM 17, a protein responsible for urinary shedding of ACE2. The enzyme activity and protein expression of ACE2 were both attenuated by administration of insulin, rosiglitazone and metformin, agents known to correct hyperglycaemia. Collectively, the studies suggested urinary ACE2 as an indicator glucose metabolic status among the diabetics.

Renin-angiotensin system blockade affected the expression of urine ACE2 in different ways. Although many studies reported no effect of RAS blockers on urinary ACE2, some studies reported significant increase and decrease in expression of urine ACE2 caused by RAS blockers. RAS blockers significantly decreased the urine expression of ACE2 protein and enzyme activity among the hypertensive diabetics but not the normotensive ones. Olmesartan, an ARB was also reported to increase both enzyme activity and ACE2 protein by a mechanism not yet well explained.

In light of these mixed findings therefore, the effect of RAS blockers on expression of urinary ACE2 remains inconclusive. As such, the findings of this survey do not suggest urine ACE2 as a potential parameter for assessing therapeutic effectiveness of RAS blockade. The effect of RAS blockers of urinary ACE2 should be further studied.
